# Seeing the similarities: Applying a coherent lens to harmful products as drivers of suicide

**DOI:** 10.1371/journal.pgph.0006437

**Published:** 2026-06-10

**Authors:** May C. I. van Schalkwyk, Duleeka Knipe, Rebecca Cassidy, Keith Tyrell, Jeff Collin, Michael Eddleston

**Affiliations:** 1 Centre for Pesticide Suicide Prevention, University of Edinburgh, Edinburgh, United Kingdom; 2 Global Health Policy Unit, School of Social and Political Science, University of Edinburgh, Edinburgh, United Kingdom; 3 Addressing the Commercial Determinants of Health in Sub-Saharan Africa (ACORDS), University of Edinburgh, Edinburgh, United Kingdom; 4 Bristol Medical School, Population Health Sciences, University of Bristol, Bristol, United Kingdom; 5 Department of Social Anthropology, University of Cambridge, Cambridge, United Kingdom; The Ohio State University, UNITED STATES OF AMERICA

## Abstract

Fatal self-harm, more commonly referred to as suicide, is a serious public health problem affecting individuals and communities worldwide. Some products and services, such as paracetamol, bridges, and railways, are recognised as requiring regulatory or design interventions to prevent suicide. However, others with well-established associations with suicide, including alcohol, gambling products, pesticides, opioids and firearms, are often framed as safe when used appropriately, even in the absence of effective regulation. In such cases, responsibility for harm is shifted onto individuals rather than directed towards harmful products and the industries that produce them. This article aims to examine this divergence, asking why certain products are problematised while others with clear links to suicide are heavily promoted and widely accessible. It examines the structural and commercial factors that sustain these inconsistencies. In so doing, we argue that such diverging approaches to harmful products deserve more effective scrutiny and call for the consistent application of a prevention lens across all products linked to self-harm and fatal outcomes. Such an approach is essential to advancing global suicide prevention efforts.

Every year over 720,000 people die by fatal self-harm, or suicide [1]. Suicide is the third leading cause of death among 15–29-year-olds globally, and the majority of suicide occurs in low- and middle-income countries (LMICs) [1]. The WHO African Region has the highest suicide rate among males compared to the global average, while the South-East Asia region shows elevated levels among females [1]. This loss of life is largely preventable [2]. In many contexts, substantial progress has been made in reducing suicide rates by acting on known drivers of lethal outcomes following self-harm [3]. However, the extent and speed of this progress are undermined by an incoherence in whether and how products, their design and regulation, are conceptualised as drivers of self-harm and fatal outcomes. This manifests as a willingness to recognise some products as inherently harmful and to regulate their design or accessibility (e.g., bridges and pharmaceuticals), while by contrast others are seen as safe when used “appropriately” (e.g., pesticides, and firearms). Here, people - and their so-called “misuse” of products - are framed as the problem. This is operationalised, for example, through the creation and cultivation of categories of people, such as “opioid abusers”, “pesticide misusers”, “problem gamblers” and “irresponsible drinkers” [4–6].

But why apply one lens to some products related to suicide and yet not to comparable others? Here, we use the concept of a product to refer to both consumer goods (e.g., alcohol and firearms) and physical environments or infrastructure (e.g., bridges and railways). We also adopt the term “self-harm” rather than suicide because many acts of self-harm with no/low suicidal intent are translated to suicide deaths when the commonly employed means are lethal (e.g., pesticides, firearms), meaning a distinction between self-harm and suicide often cannot be made [7]. When we use the term self-harm, we are referring to both fatal and non-fatal self-harm, unless otherwise indicated.

This article examines the varying willingness to apply a prevention lens to risk factors (e.g., gambling) that increase the risk of self-harm and to lethal means (e.g., firearms) that influence the likelihood of survival of the self-harm attempt. In so doing, it aims to problematise the incoherence across drivers of self-harm and to consider whose interests are ultimately served by a given way of thinking about the interplay among products, self-harm and its prevention. To achieve this, we focus on key products where there is clear evidence of their contribution to the global burden of fatal self-harm and of powerful commercial vested interests in their continued promotion, accessibility, and sale.

## Manufacturing difference

Effective suicide prevention measures recognise that fatal self-harm commonly occurs impulsively during short-lived crises or overwhelming moments of distress [8]. The vast majority of those who engage in self-harm (regardless of intent) survive and most will not go on to die by suicide [9,10]. Put simply, if highly lethal means of self-harm are not readily accessible and/or points of resistance can be built-in to slow actions down, many more self-harm events would not result in death. If lived environments are safe, people can be given the chance to make it through these moments in their lives, and survive with effective support from community or healthcare services [8]. This logic underpins the design of physical barriers on bridges, other high structures, and subway/rail networks, for example, as well as the mandating of blister packs, small pack sizes, and restrictions on the number of packets of medicines that can be sold within a single purchase or given period [11]. The former reduces the risk of fatal self-harm created by access to lethal heights or subway/rail tracks, while the latter aims to prevent impulse purchasing and consumption of lethal doses of pharmaceuticals. These measures have a well-established evidence-base of effectiveness [11–13].

However, such measures are not universally adopted and at times have even been withdrawn with devasting effects. For example, following the removal of the barriers on a bridge in New Zealand, a fivefold increase in the number and rate of fatal self-harm from the bridge was observed [14]. Barriers of an enhanced design were reinstalled and no fatal self-harm occurred from the bridge in the subsequent three year period [14]. Similarly, prevention measures may be adopted but in a weaker form. For example, in the UK, some policies concerning the sale of medicines for pain relief, such as paracetamol, are not enshrined in law. Instead, the Medicines and Healthcare Products Regulatory Agency issues voluntary guidance advising retailers to not “sell more than two packs in any one transaction” or “use offers that encourage the sale of more than one pack” [15]. This self-regulatory arrangement means retailers can breach the guidance but still be acting within the law, leading to calls for a legislative ban on multi-buy deals of paracetamol in order to “protect public health and prioritise patient safety over profit” [16].

Whilst acknowledging both the absence of similar interventions in many places and their limitations, the promotion and implementation of such measures by decisionmakers are important in signalling a willingness to problematise and regulate products to prevent self-harm. Across all these measures, the implicit underlying logic is that some products act as drivers of fatal self-harm and should therefore be designed and restricted in ways that prioritise preventing these deaths. Yet this lens is not consistently applied to all products associated with an increased risk of fatal self-harm, which raises the question: why some products? And not others? Are these contrasts systematic? What are the barriers to achieving consistency across all products associated with an increased risk of fatal self-harm, and how might they be overcome?

Radically different approaches are often taken towards products that contribute to the global burden of fatal self-harm, notably: alcohol, gambling, opioids, firearms, and pesticides [5]. Despite being inherently harmful, in many contexts the framing advocated by industry and policymakers is that these products – even in their most dangerous designs or formulations – can safely be used among communities, even in the absence of effective regulation. Suicide or fatal self-harm is not routinely listed as a potential product harm or seen to potentially result from the design, marketing, or accessibility of these products. Instead, fatal self-harm is understood to be a problem that emerges because a person “misuses” or “abuses” these products [5,6,17,18]. People’s choices and behaviours then become the focus of research and intervention. In the following sections we examine how this thinking creates paradoxical situations in which people are expected to keep themselves and their loved ones safe while living in unsafe environments.

We begin by examining products the use of which is associated with an increased risk of self-harm, namely alcohol and gambling products, follow by an examination of opioids, firearms and pesticides, which increase the risk of fatal self-harm or suicide. In doing so, we draw on country-level examples that were informed by the collective experiences and expertise of the authors, and the evidence base on the corporate political activities of the included industries. However, these should be viewed as illustrative and do not represent an exhaustive collection of all relevant regulatory contexts and commercial products and practices. These examples are also intended to be interpreted as different manifestations of what we argue is a universal systemic problem where, beyond the range of products contributing to the risk and burden of suicide in a given context, an inconsistent framing and policy approach is overwhelmingly the norm rather than the exception.

### Gambling and alcohol

Gambling and use of alcohol, both acute intoxication and long-term drinking, are associated with an increased risk of self-harm and suicide [19–21]. The WHO estimates that 22% of all fatal self-harm can be attributed to alcohol use and advises that restrictions on alcohol availability and marketing as well as pricing policies represent important elements of suicide prevention strategies [2]. The implementation of alcohol restrictions is associated with reduced fatal self-harm. For example, reductions in male fatal self-harm in Russia and in fatal self-harm rates in South Africa were observed after the introduction of the 2006 Russian alcohol policy and covid-19 alcohol restrictions respectively [22,23]. The WHO recognises that gambling can lead to serious harms to health including suicide [24]. While the public health burden of gambling is difficult to estimate due to a lack of high-quality relevant data, there are a growing number of studies documenting the burden of gambling-related suicide/fatal self-harm in both high and low income country settings [25–27]. Yet in many contexts, gambling and/or alcohol products are available 24/7 and promoted using highly sophisticated marketing strategies that glamourise and normalise their use and play down the harm to consumers and others. Reminiscent of the tobacco industry’s elaborate marketing strategies [28,29], major alcohol and gambling companies use celebrity and sports sponsorships to drive brand awareness, engagement and sales [30,31]. They target specific groups, such as women or young people, through tailored marketing and product designs and/or employ innovative strategies, including surrogate (promotion of different, permissible products using the same branding as the restricted product) and alibi (use of branding cues or slogans that likely remind consumers of the restricted product in the absence of explicitly mentioning its name) marketing to circumvent advertising restrictions [32–34]. These industries act to influence public health policymaking [35,36], and continue to aggressively expand their markets, particularly in LMICs [37,38].

Both the gambling and alcohol industries derive a substantial amount of their profits from heavy consumers [24,39] and actively oppose measures intended to prevent the harms associated with their products [35]. Unlike other consumer products, gambling products are not universally tested for safety [40], despite being designed to keep people playing to “extinction” (i.e., until their money runs out) [41], and neither gambling nor alcohol products come with mandated warnings about the links between these products and the risk of self-harm and suicide.

Warning and informing the public about the harms associated with these products is often left by government to industry or their funded organisations, despite the conflicts of interest this creates. In documented cases, these organisations claim to be independent of their industry funders and evidence-based while disseminating misinformation and promoting industry-favourable framings about who is to blame and what is needed to prevent harm [17,42,43]. Many of their purported public health campaigns and youth education prevention programmes promote the largely discredited rhetoric of responsible drinking and gambling [17,42,43]—consumption patterns that are completely undermined by the marketing and (mis)information people are exposed to, and the norms embedded by industry and government policy. The public bear the burden of navigating unsafe and highly liberalised alcohol and gambling markets and are then labelled as irresponsible or inherently faulty when harm arises. When prevention measures are adopted, this is often in an incoherent, incremental way and does not involve removing dangerous product(s) from the market. For example, in the UK, the gambling industry is tasked by the regulator with preventing harm by monitoring people for signs of harm—the same characteristics that define their most profitable customers [40]. This does not make sense and there is no evidence that this is effective as a suicide prevention measure.

### Opioids

The liberalisation of access to prescription opioids in the US, which fuelled a deadly crisis, similarly rested on defining people and a supposedly out-of-date and unnecessarily hesitant medical community as the problem [18,44]. The crisis resulted in hundreds of thousands of preventable deaths including fatal self-harm [45,46]. The relationship between opioids and fatal self-harm is multifaceted. First, opioid use disorder, much of which has been fuelled in the US crisis by escalating prescription of high-strength opioids, increases the risk of fatal self-harm as does suffering from chronic pain [47,48]. Second, evidence suggests that access to opioids increases the likelihood that a self-harm episode will result in death; a similar relationship has been observed between the consumption of alcohol and the likelihood and lethality of self-harm [45,49]. A US study estimated that 34% and 81% of self-harm events involving specific drugs would not have been fatal if alcohol and opioids respectively were not involved [45].

The opioid crisis was clearly driven by the strategies of the opioid industry, which played down the risk of addiction and redefined established medical practice with regards to pain management in ways favourable to their business interests [44,50]. The opioid industry and those in receipt of its funding legitimised the prescription of escalating doses of potent opioid-based drugs by redefining people, and their so-called misuse or abuse of opioids, as the problem [18]. They constructed concepts like *pseudo-addiction* to justify continued prescribing of opioids and *opioid-phobia* to present concerns raised within the medical community as unfounded and based on old-fashioned thinking [44]. This framing obscured the role played by the industry, the design and marketing of its products, weaknesses within the pharmaceutical supply chain, and the misuse of data and scientific evidence [50]. Recognition of the role played by the pharmaceutical industry and the failure of the regulatory and health systems to prevent industry influence and aggressive promotion in the pursuit of profits, led the Lancet Commission on *Responding to the opioid crisis in North America and beyond* to recommend curtailing pharmaceutical product promotion and dismantling the industry’s influence on universities and professional bodies, regulators, and politics [46]. Focusing on the product and its producers is a necessary first step to avert future crises, and in the context of products like opioids, addresses both the increased risk of fatal self-harm and access to lethal means.

### Firearms

The US also provides an important example in relation to firearm related fatal self-harm and widespread access to such highly lethal means. The US has one of the highest rates of firearm deaths globally [51], a substantial proportion of which are due to fatal self-harm [52]. More people die from firearm self-harm than all other methods combined, and access to firearms is a risk factor for fatal self-harm [ 53,54] . Living in a home with a firearm substantively increases the risk of fatal self-harm among adolescents [55]. A relationship between alcohol use and firearm related fatal self-harm has also been observed, with evidence linking increasing alcohol consumption with a heightened risk of firearm related fatal self-harm [56].

Despite the overwhelming evidence linking firearm access and availability with increased deaths due to fatal self-harm and other forms of injuries, such as homicide, measures that take as a given the presence of firearms in homes and communities are prioritised. This approach is in part legitimised by framing people and their mental health as the problem. This places the onus on individuals and families to ensure that people at risk of self-harm do not have access to firearms. This is to be achieved, for example, by adopting ‘safe storage’ measures or removing a firearm from the home until such time as the person is no longer at risk [57].

This type of risk management is favoured by members of the firearm industry [5]. It leaves the structural drivers of fatal self-harm in place: widespread accessibility of highly lethal products designed to cause penetrating injuries. Representatives of the firearm industry partner with reputable suicide prevention charities to promote ‘safe storage’-based initiatives and have been shown to make unsupported claims about the impacts of such programmes [58,59]. The firearm lobby promotes firearm ownership as a means of protecting one’s safety and the American way of life, while distorting the evidence base and opposing restrictions on firearm accessibility and design [60–62]. As with the gambling industry, the firearm industry and its products have been granted immunity from the types of mandatory safety testing and standards to which other consumer products are subject [63].

### Pesticides

Finally, the case of pesticides is particularly notable given that pesticide self-poisoning is one of the leading means of fatal self-harm globally, with striking evidence in support of banning acutely toxic highly hazardous pesticides (HHPs) as a form of means restriction to prevent fatal self-harm [64]. The considerable burden of harm caused by HHPs is completely preventable and strikingly unethical. The idea that more pesticides are essential to meeting the agricultural needs of the world’s population – as promoted by the pesticide industry – has been described by the United Nations’ special rapporteur on the right to food as “a myth” and “dangerously misleading” [65]. Yet pesticide producers continue to profit from the sale of toxic pesticides known to be associated with death of users as well as fatal self-harm [66].

Some of these products are manufactured in high-income countries, where they have been banned domestically because of their hazardous characteristics, for export and use in LMICs which have the greatest burden of fatal self-harm due to pesticide self-poisoning [66]. Members of the pesticide industry justify this export of highly toxic products by claiming it is responding to the unique agricultural needs of importing sovereign countries [67]. This conflicts with the views of those who have lost someone to pesticide related fatal self-harm and with the evidence that the banning of HHPs can be achieved without impacting agricultural productivity [67,68]. For example, Sri Lanka implemented a series of successive bans of pesticides identified as being responsible for the majority of pesticide deaths. These regulations are believed to have contributed to one of the most substantial reductions in fatal self-harm rates ever observed, and in the absence of any apparent detrimental impacts on agricultural yield [69]. The export of banned pesticides from the Global North, of which the UK and the EU are major players, to the Global South has been described by the United Nations’ special rapporteur on toxics and human rights as a form of “modern-day exploitation” [70]. Addressing the global toll of lethal pesticide self-poisoning requires that this practice is ended alongside a rapid phase out of HHPs globally.

However, the pesticide industry and other actors claim that HHPs are safe if used in the “correct” way [6]. This conflicts with a hazard-based approach, adopted by the EU for example, which recognises that it is not feasible to establish a safe way of using HHPs within communities and that banning their production, sale and use is the only effective way to achieve safety [6]. Unfortunately, other means of restricting access to them, such as ‘safe storage’, which are not supported by the independent evidence-base [71], have been promoted as alternatives to bans [72]. These measures frame people, usually farmers and their families from poor and rural communities, and their “misuse” of products as the problem, not the ongoing accessibility of HHPs [6]. At an international level, this framing is echoed by, for example, the Rotterdam Convention which governs elements of the international trade of hazardous chemicals and pesticides. The convention does not permit the use of evidence for a pesticide’s contribution to fatal self-harm to inform its proceedings [ 6,73] . Specifically, the convention explicitly states that “intentional misuse is not in itself an adequate reason to list a chemical” among those that have been banned or restricted by member states [ 6,73].

In summary, looking across these examples, people are being burdened and blamed while harmful products are heavily promoted and framed as safe in ways that obscure their hazardous or addictive properties and the real-world contexts in which they are marketed, used and the resultant impacts. Preventing fatal self-harm will entail effectively curtailing the production, trade, and widespread accessibility of highly lethal means and creating safe and healthy environments by restricting the practices of commercial actors that serve to promote the deregulation and use of harmful products known to increase the risk of fatal self-harm. However, achieving such profound shifts in policy and practice rests on answering some pressing questions and adopting new ways of thinking.

### The urgent need for change

So why one approach for bridges and another for pesticides? Why are addictive gambling products more accessible than paracetamol? Why are most consumer products but not guns tested and regulated for safety? Why are HHPs still in use when other far less lethal and more sustainable products and methods are available? The answers lie in the varied social histories of these products including the extent of regulatory capture. To answer these urgent questions, researchers and practitioners working collaboratively across fields and disciplines need to use a range of methods and approaches to ask who is ultimately benefitting from a willingness to problematise some products and not others and, more importantly, how these entrenched advantages might be disrupted.

Different ways of thinking about self-harm/suicide and its drivers are both possible and urgently needed. We do not wish to undermine the importance of recognising that not every self-harm episode is driven by the same set of factors. Nor do we believe that access to the products discussed here explains all fatal self-harm. Our argument is that diverging approaches to harmful products deserve more effective scrutiny as they have implications for preventing tens of thousands of deaths every year. While it may seem intuitively appealing to promote downstream individual-level risk management practices as a pragmatic response to what may seem like intractable policy conflicts or limited resources, we must guard against presenting less or ineffective or impractical interventions as capable of preventing suicides whilst leaving the drivers of those suicides in place. Rejecting logics that are highly favourable to the interests of some commercial sectors that should instead be seen as important drivers of fatal self-harm is critical to preventing suicide the world over. To save lives, products that are unnecessarily unsafe and harmful must be viewed with the same lens.
